# Analysis of *OTX2*, *PAX6*, and *SOX2* Gene and Protein Expression Patterns in Ocular Development of Human and Rat Embryos

**DOI:** 10.3390/ijms262210845

**Published:** 2025-11-08

**Authors:** Anna Junga, Mara Pilmane, Pavlo Fedirko

**Affiliations:** 1 Institute of Anatomy and Anthropology, Riga Stradins University, LV-1010 Riga, Latvia; mara.pilmane@rsu.lv; 2 Institute of Radiation Hygiene and Epidemiology, 04050 Kyiv, Ukraine; eye-rad@ukr.net

**Keywords:** eye development, genes, proteins, histology, in situ hybridization, immunohistochemistry

## Abstract

Transcription factors orthodenticle homeobox 2 gene (*OTX2*), paired box 6 gene (*PAX6*), and SRY-box transcription factor 2 gene (*SOX2*) are key regulators of ocular morphogenesis; however, their comparative embryonic localization across species—and the correspondence between transcript and protein distributions—remain incompletely defined. Chromogenic in situ hybridization (CISH) was employed to detect *OTX2*, *PAX6*, and *SOX2* transcripts, while biotin–streptavidin immunohistochemistry (IHC) was used to assess Otx2, Pax6, and Sox2 protein expression. A semi-quantitative scoring system was applied to evaluate positive structures across ocular compartments. Transcripts were predominantly localized to the retina in both species, with occasional low-level expression in the optic nerve, sclera, and eyelid. Proteins displayed broader distributions: Otx2 was abundant in the retina and eyelid, while Pax6 and Sox2 were detected in multiple tissues, including cornea and extraocular muscles. *OTX2*, *PAX6*, and *SOX2* show retina-predominant transcription and wider protein expression across ocular tissues. These findings highlight spatial differences between transcript and protein localization, supporting a complex regulatory framework underlying vertebrate eye development.

## 1. Introduction

Although eye formation has been extensively investigated, many aspects remain incompletely understood. In mammals, ocular morphogenesis begins with an evagination of the early neural plate prior to neural tube closure [1]. Recent single-cell atlases and spatial datasets of the developing human retina now resolve progenitor-to-neuron trajectories, regional patterning (e.g., macula vs. periphery), and underlying regulatory programs, thereby refining the timing and lineage relationships that underlie optic cup formation [2,3,4]. These advances provide a contemporary framework that integrates classical morphogenesis with cell-state-resolved and organoid-derived insights, positioning the present study within current efforts to map ocular development at single-cell resolution. Key regulators of this process include transcription factors from the orthodenticle homeobox (*OTX*), paired box (*PAX*), and SRY-box (*SOX*) gene families, which coordinate tissue specification, morphogenesis, and cell differentiation.

The mammalian genome contains three *OTX* genes: *OTX1*, *OTX2*, and the divergent Crx [5]. Among them, OTX2 encodes a homeobox-containing transcription factor essential for head and sensory organ development [6]. It regulates the formation of the forebrain, midbrain, pineal and pituitary glands, as well as the inner ear and visual system [7,8,9,10,11]. In the eye, *OTX2* is expressed during optic vesicle stage and is required for retinal pigment epithelium (RPE) specification, photoreceptor differentiation, and bipolar cell maturation [10]. In the mature retina, *OTX2* persists in bipolar, photoreceptor, and RPE cells, which together maintain visual function [8,12,13]. Mutations in *OTX2* lead to a spectrum of ocular malformations, including anophthalmia, microphthalmia, coloboma, and retinal dystrophies, sometimes accompanied by hypopituitarism [6,11,14]. These findings highlight the critical and dosage-sensitive role of *OTX2* in ocular development (reviewed in [15]).

The *PAX6* gene, located on chromosome 11p13, is widely regarded as a master regulator of eye development and also plays an essential role in development of the central nervous system (CNS) and pancreas. Despite its broad range of functions, *PAX6* mutations most often manifest as ocular abnormalities, particularly iris malformations [16]. *PAX6* is expressed from the earliest stages of ocular morphogenesis, where it governs optic vesicle formation, lens induction, epithelial morphogenesis, and retinal cell specification [17,18]. Mutations in *PAX6* result in structural macular defects, foveal hypoplasia, retinal layer thinning, and aberrant retinal development, often leading to severe vision impairment [19]. Coding-region mutations typically give rise to the most severe phenotypes, including aniridia and optic nerve anomalies [20]. *PAX6* function is highly dosage-sensitive, with both under- and overexpression impairing ocular morphogenesis [21]. Beyond its role in oculogenesis, *PAX6* also regulates lineage specification and neuronal differentiation within the CNS, frequently cooperating with *SOX2* to control brain patterning and cortical development [22,23]. Acting in concert with *OTX2* and *SOX2*, *PAX6* participates in the “eye-field transcription factor network,” which orchestrates early eye-field specification and ensures coordinated development of ocular structures.

The third factor, *SOX2*, is a highly conserved transcription factor crucial for embryogenesis, stem cell maintenance, and tissue-specific differentiation. Originally identified as a marker of neural stem cells, *SOX2* has been extensively studied in CNS development, including retinal morphogenesis from the neuroectoderm [24]. It preserves the self-renewal capacity of neural progenitors and regulates the balance between proliferation and differentiation during neurogenesis [24,25]. Mutations in *SOX2* are associated with severe ocular malformations, particularly bilateral anophthalmia and microphthalmia, with about 10% of cases due to *SOX2* haploinsufficiency [25,26]. In the retina, *SOX2* is expressed in multipotent retinal progenitor cells and later in Müller glia, amacrine, and ganglion cells [25,27]. Its expression must be tightly regulated, as altered *SOX2* levels lead to disrupted retinal cytoarchitecture, impaired neuronal connectivity, and vision loss [25,27,28]. A decline in *SOX2* expression with aging correlates with reduced Müller glia activity, diminished neuronal support, and progressive vision impairment [25]. Functionally, *SOX2* promotes amacrine cell differentiation through induction of PAX6, whereas its suppression reduces cellularity of the inner nuclear layer [27]. The Sox2 protein itself is subject to multiple post-translational modifications, including phosphorylation, acetylation, and ubiquitination [29]. In addition to its developmental and homeostatic functions, *SOX2* has been implicated in tumorigenesis, being frequently amplified or overexpressed across various cancers, including glioblastoma, medulloblastoma, and several epithelial malignancies [30].

This study aimed to characterize the expression patterns of *SOX2*, *PAX6*, and *OTX2* genes and proteins in the context of eye morphogenesis. It was hypothesized that, although these transcription factors share overlapping roles in ocular development, their transcript and protein distributions differ across ocular tissues and species, reflecting distinct spatial levels of post-transcriptional regulation.

## 2. Results

### 2.1. Expression of OTX2 Gene and Protein in Human and Rat Embryonic Eyes

**Figure 1:**
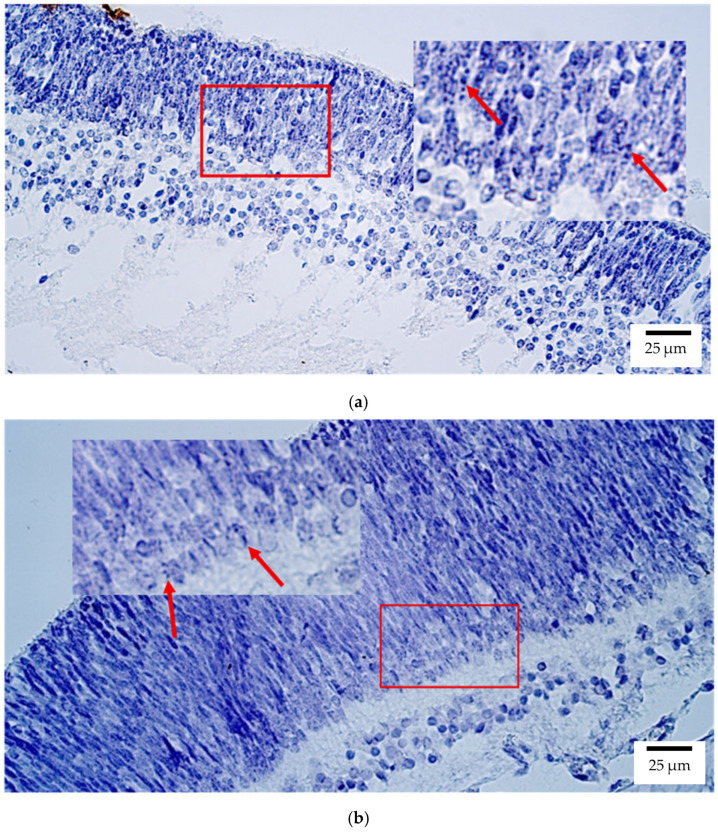
Gene amplification levels of *OTX2* gene were visualized using chromogenic in-situ hybridization (CISH) in (**a**) human and (**b**) rat embryos. (**a**) Moderate to numerous (++/+++) gene copies in retina of a 7th–8th week human embryo (Carnegie stage 23). *OTX2* CISH, ×400. (**b**) Moderate (++) gene copies in retina of a 2nd–3rd week rat embryo. *OTX2* CISH, ×400. The red arrow shows the localization of the gene probe hybridization signals.

Chromogenic in situ hybridization (CISH) analysis revealed that *OTX2* expression in human embryonic eyes was predominantly localized within the retina, where a median number of positive structures was detected at levels ranging from moderate to numerous (++/+++) and numerous (+++) (Figure 1a). A few to moderate (+/++) and moderate (++) expression levels were observed in the optic nerve, sclera, and eyelid, whereas other ocular tissues, including the lens, ciliary body, iris, cornea, choroid, and eye muscles, showed minimal or no detectable signal.

In rat embryos, *OTX2* was similarly localized mainly to the retina, where a moderate number (++) of positive structures was observed (Figure 1b). Low expression (few positive structures (+)) was detected in the optic nerve, sclera, and eyelid, while no signal was detected in the lens, ciliary body, iris, cornea, choroid, or eye muscles.

**Figure 2:**
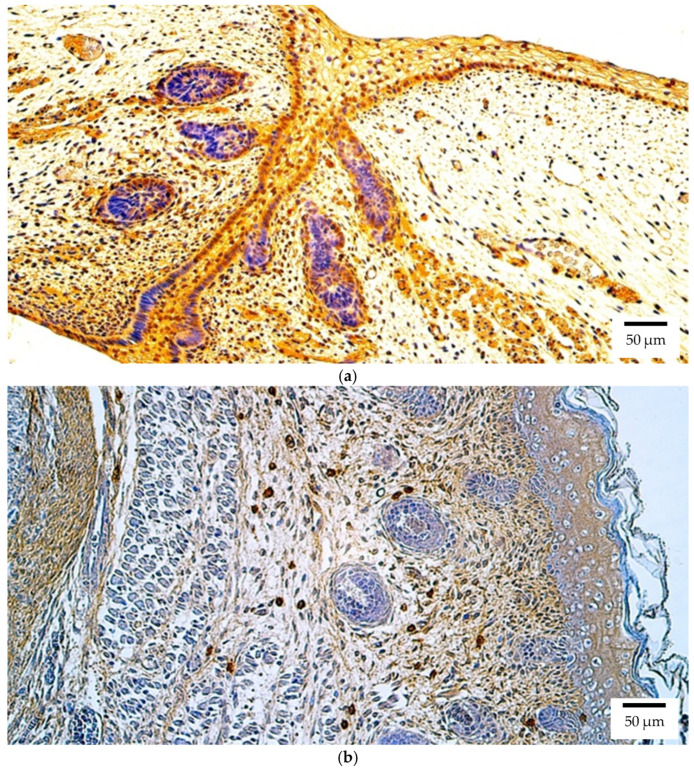
Otx2 protein expression detected by immunohistochemistry (IHC) in (**a**) human and (**b**) rat embryos. (**a**) Abundant positive (++++) epithelial cells, connective tissue cells, and skeletal muscle precursor cells are visible in the eyelid of a 6th–7th week human embryo (Carnegie stage 18–22). Otx2 IHC, ×200. (**b**) A moderate number of positive (++) connective tissue cells is visible in the eyelid of a 2nd–3rd week rat embryo. Otx2 IHC, ×200.

In contrast, immunohistochemical analysis of Otx2 protein revealed a broader distribution. The most prominent staining was detected in the human retina and eyelid, both showing abundant positive structures (++++) (Figure 2a). All other ocular tissues, including the lens, optic nerve, ciliary body, iris, cornea, sclera, choroid, and eye muscles, exhibited a few positive structures (+). This pattern indicates that the Otx2 protein is more widely distributed than its transcript in the human embryonic eye (lens, optic nerve, and iris: *p* = 0.014; cornea, choroid, and eye muscles: *p* = 0.020; eyelid: *p* = 0.042)

At the protein level in the rat embryo, Otx2 immunoreactivity was again most pronounced in the retina and eyelid, both demonstrating numerous positive structures (+++) (Figure 2b). In the lens, optic nerve, ciliary body, iris, cornea, sclera, and choroid, Otx2 protein was present at low levels (few positive structures (+)). Eye muscles were negative for Otx2.

Statistically significant differences between human and rat embryo groups were not observed. All results are summarized in Supplementary Table S1.

**Figure 3:**
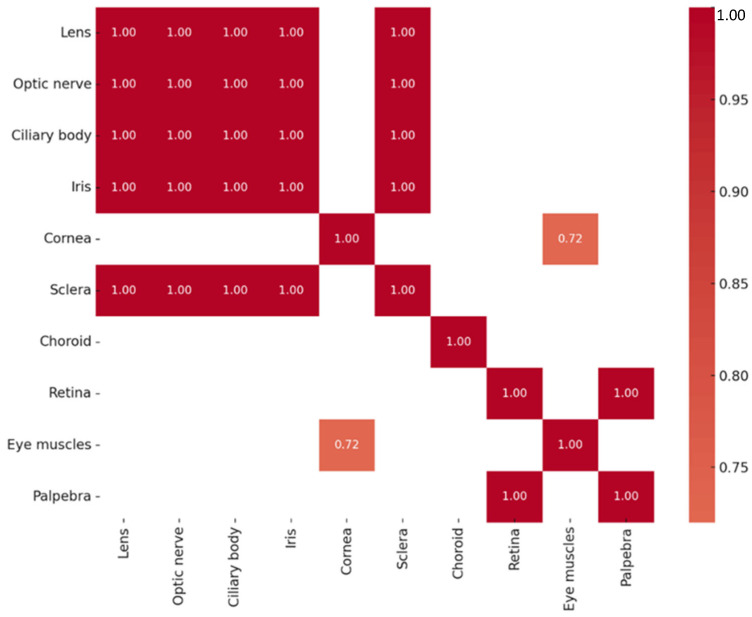
Heatmap of statistically significant Otx2 protein correlations.

A series of highly significant correlations (r_s_ = 1.000, *p* < 0.01) were identified among Otx2 protein levels across multiple ocular tissues, including the lens, optic nerve, ciliary body, iris, sclera, and eyelid. A strong correlation was also detected between the cornea and the extraocular muscles (r_s_ = 0.720, *p* = 0.029). The correlations of Otx2-positive structures are summarized in Figure 3. Across all tissues analyzed, Otx2 protein expression did not exhibit statistically significant correlation with *OTX2* gene expression.

### 2.2. Expression of PAX6 Gene and Protein in Human and Rat Embryonic Eyes

**Figure 4:**
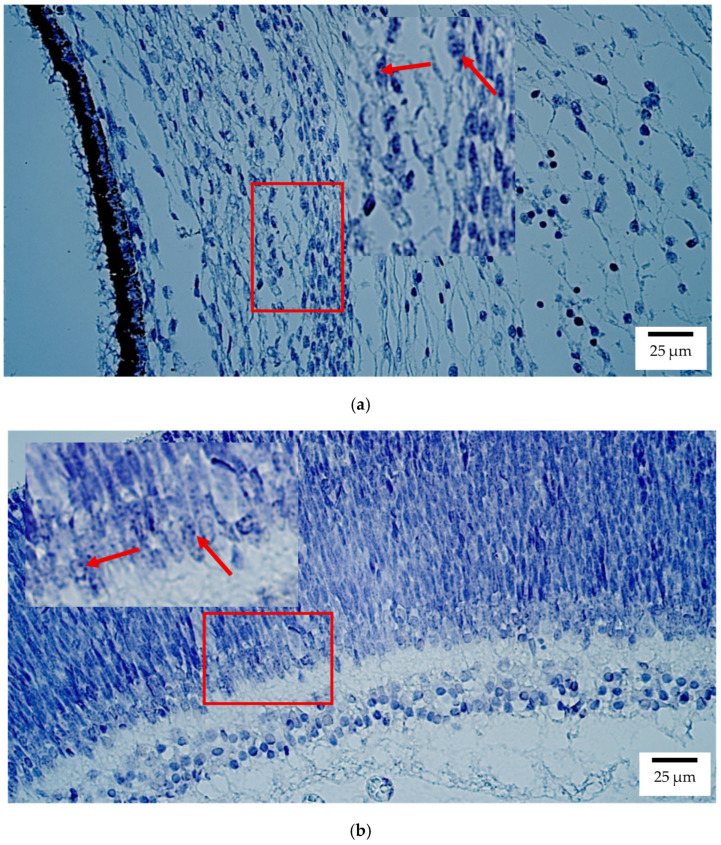
Gene amplification levels of *PAX6* gene were visualized using chromogenic in-situ hybridization (CISH) in (**a**) human and (**b**) rat embryos. (**a**) Few to moderate (+/++) gene copies in sclera of an 8th–9th week human embryo (Post-Carnegie stage). *PAX6* CISH, ×400. (**b**) Moderate (++) gene copies in retina of a 2nd–3rd week rat embryo. *PAX6* CISH, ×400. The red arrow shows the localization of the gene probe hybridization signals.

In human embryonic eyes, CISH analysis revealed that *PAX6* expression was most prominent in the retina, with levels ranging from moderate (++) and moderate to numerous (++/+++) positive structures. A few (+) to few-to-moderate (+/++) numbers of positive structures were also present in the optic nerve, sclera (Figure 4a), and eyelid, whereas other ocular tissues, including the lens, ciliary body, iris, cornea, choroid, and eye muscles, were negative.

In rat embryos, *PAX6* was again highest in the retina (++) (Figure 4b), with low expression (+) in the optic nerve, sclera, and eyelid. The lens, ciliary body, iris, cornea, choroid, and eye muscles showed no detectable transcripts.

**Figure 5:**
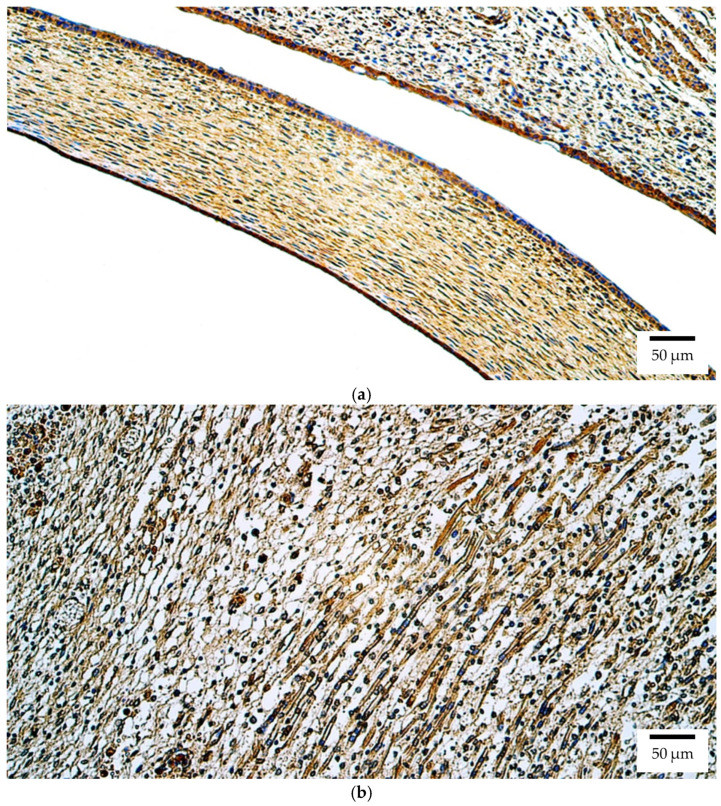
Pax6 protein expression detected by immunohistochemistry (IHC) in (**a**) human and (**b**) rat embryos. (**a**) Numerous positive (+++) epithelial and connective tissue cells are visible in the cornea and eyelid of a 6th–7th week human embryo (Carnegie stage 18–22). Pax6 IHC, ×200. (**b**) Numerous positive (+++) skeletal muscle precursor cells are visible in the developing extraocular muscles of a 2nd–3rd week rat embryo. Pax6 IHC, ×200.

Immunohistochemistry demonstrated that Pax6 protein in human embryos was broadly distributed, with numerous (+++) positive structures observed in the lens, optic nerve, ciliary body, iris, retina, eye muscles, and eyelid (Figure 5a). The cornea exhibited moderate-to-numerous (++/+++) to numerous (+++) positive structures (Figure 5a), while the sclera and choroid displayed moderate (++) expression levels.

At the protein level in rat embryos, Pax6 was observed with numerous (+++) positive structures in the retina, eye muscles (Figure 5b), and eyelid. A moderate (++) level of expression was observed in all other ocular tissues.

Statistically significant differences between human and rat embryo groups were not observed. All results are summarized in Supplementary Table S1.

**Figure 6:**
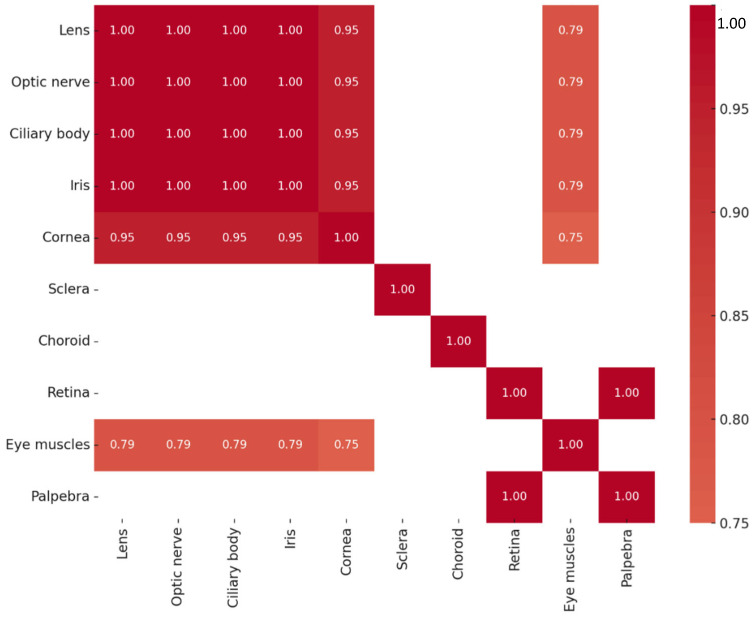
Heatmap of statistically significant Pax6 protein correlations.

Multiple strong and statistically significant correlations in Pax6 protein expression were observed among ocular tissues. Perfect correlations (r_s_ = 1.000, *p* < 0.01) were found between the lens, optic nerve, ciliary body, and iris, as well as between the retina and eyelid. Similarly, very strong correlations were observed among the lens, optic nerve, ciliary body, iris, and cornea (r_s_ = 0.949, *p* < 0.01). Strong correlations were also identified between the extraocular muscles and several tissues, including the optic nerve, lens, ciliary body, and iris (r_s_ = 0.791, *p* = 0.011 for each pair), as well as between the cornea and extraocular muscles (r_s_ = 0.750, *p* = 0.020). The correlation patterns of Pax6-positive structures are summarized in Figure 6. In contrast, no statistically significant correlations were detected between Pax6 protein and gene expression levels across tissues.

### 2.3. Expression of SOX2 Gene and Protein in Human and Rat Embryonic Eyes

**Figure 7:**
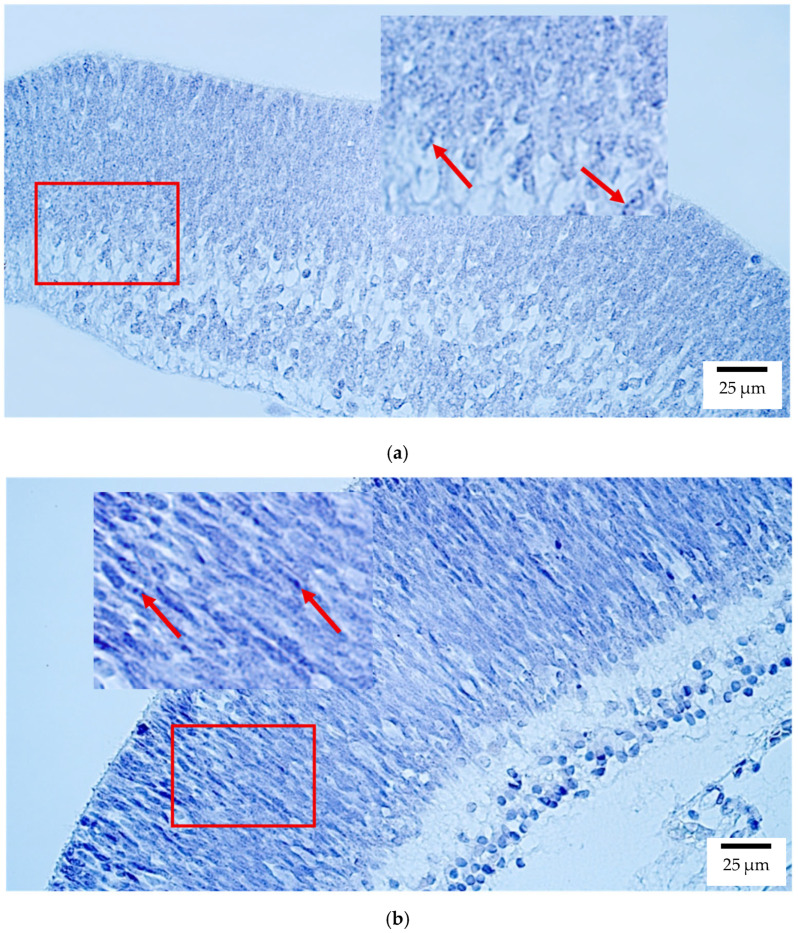
Gene amplification levels of *SOX2* gene were visualized using chromogenic in-situ hybridization (CISH) in (**a**) human and (**b**) rat embryos. (**a**) Moderate (++) gene copies in retina of a 7th–8th week human embryo (Carnegie stage 23). *SOX2* CISH, ×400. (**b**) Moderate (++) gene copies in retina of a 2nd–3rd week rat embryo. *SOX2* CISH, ×400. The red arrow shows the localization of the gene probe hybridization signals.

CISH showed that *SOX2* transcripts were mainly localized to the retina in both human and rat embryos, with moderate to numerous (++/+++) expression (Figure 7a,b). Few to moderate (+/++) signals were detected in the optic nerve, sclera, and eyelid, whereas the lens, ciliary body, iris, cornea, choroid, and extraocular muscles lacked detectable transcript expression.

**Figure 8:**
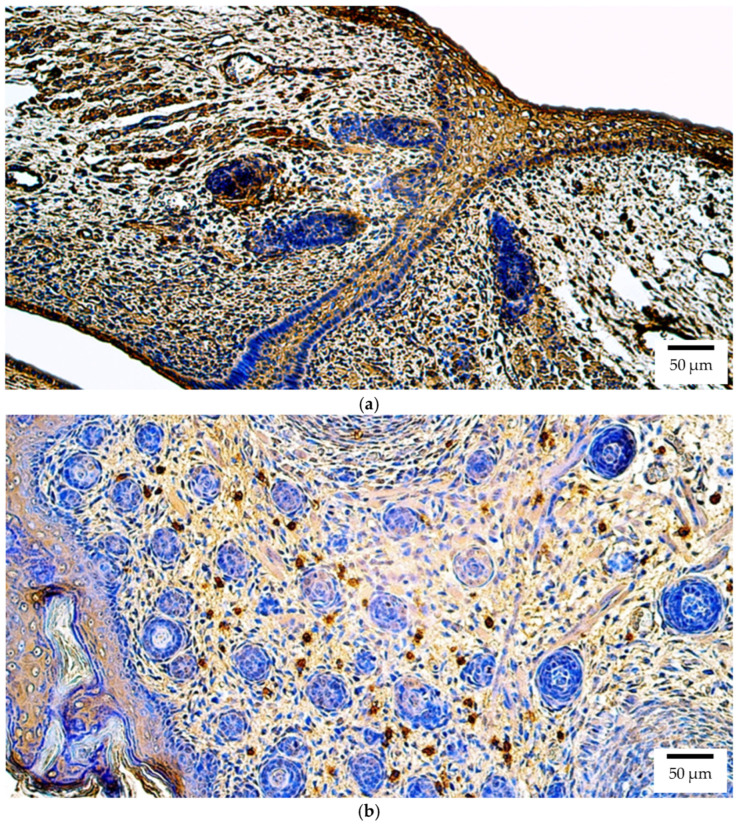
Sox2 protein expression detected by immunohistochemistry (IHC) in (**a**) human and (**b**) rat embryos. (**a**) Numerous positive (+++) epithelial cells, connective tissue cells, and skeletal muscle precursor cells are visible in the eyelid of a 6th–7th week human embryo (Carnegie stage 18–22). Sox2 IHC, ×200. (**b**) A moderate number of positive (++) connective tissue cells is visible in the eyelid of a 2nd–3rd week rat embryo. Sox2 IHC, ×200.

In contrast, the Sox2 protein exhibited a broader distribution (lens, optic nerve, and iris: *p* = 0.023; cornea, choroid, and eyelid: *p* = 0.024; eye muscles: *p* = 0.026). In human embryos, numerous positive structures (+++) were observed in the retina, eyelid, and cornea (Figure 8a), and moderate to numerous (++/+++) levels in the extraocular muscles; other tissues, including the lens, optic nerve, ciliary body, iris, sclera, and choroid, showed moderate (++) expression. In rat embryos, Sox2 immunoreactivity was moderate (++) across all ocular structures (Figure 8b). No statistically significant interspecies differences were detected (Supplementary Table S1).

**Figure 9:**
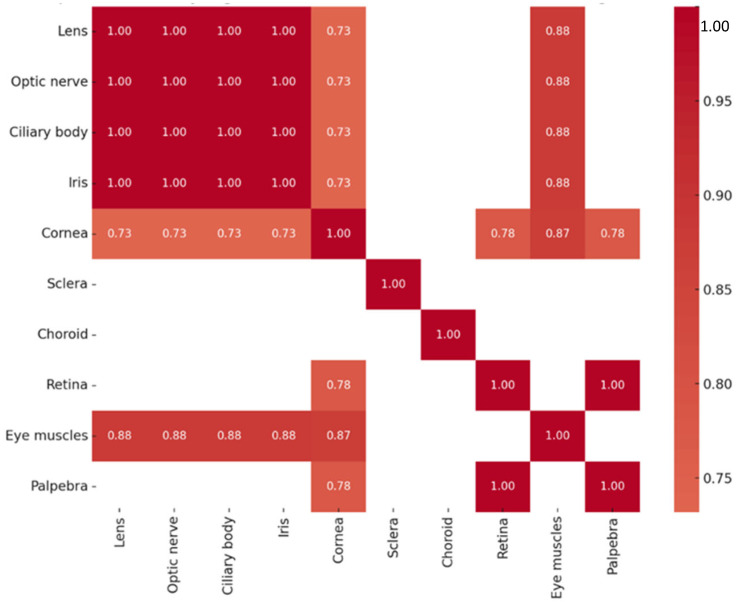
Heatmap of statistically significant Sox2 protein correlations.

Analysis of Sox2 protein levels revealed multiple strong and statistically significant correlations among ocular tissues. Perfect correlations (r_s_ = 1.000, *p* < 0.01) were observed between the lens, optic nerve, ciliary body, and iris, as well as between the retina and eyelid. Very strong positive correlations were also found between the extraocular muscles and several tissues, including the lens, optic nerve, ciliary body, and iris (r_s_ = 0.878, *p* = 0.002 for each pair). Furthermore, strong positive correlations were observed between the cornea and extraocular muscles (r_s_ = 0.871, *p* = 0.002), as well as between the cornea and other ocular tissues—including the lens, optic nerve, ciliary body, and iris (r_s_ = 0.732, *p* = 0.025 for each pair)—and with the retina and eyelid (r_s_ = 0.775, *p* = 0.014 for both). The correlations of Sox2-positive structures are summarized in Figure 9. No significant correlations were observed between Sox2 protein and *SOX2* transcript expression.

## 3. Discussion

This study provides a comparative analysis of *OTX2*, *PAX6*, and *SOX2* expression in human and rat embryonic eyes, revealing both conserved and distinct expression patterns at the transcript and protein levels.

CISH localized *OTX2* transcripts primarily to the retina in both species, with a tendency toward higher expression in humans than in rats, although this difference did not reach statistical significance. In contrast, Otx2 protein detected by IHC exhibited a broader distribution, with abundant staining in the retina and eyelid and low-level expression across most ocular tissues.

Our data support the concept that *OTX2* occupies a central position in the gene regulatory network governing retinal progenitor differentiation into photoreceptors and bipolar cells [9]. The strong retinal expression observed here is consistent with previous reports indicating that *OTX2* is essential for photoreceptor and bipolar cell specification, and that its reduction results in structural disorganization, neuronal degeneration, and progressive vision loss [8]. The regulatory role of *OTX2* extends beyond differentiation, encompassing the maintenance of cell-type identity and the control of genes required for the phototransduction pathway [8,31]. The transcript localization we observed mirrors these developmental roles, while the broader protein distribution suggests that *OTX2* stability or translational control ensures its persistence beyond sites of active transcription.

The discrepancy between transcript restriction and widespread protein distribution is particularly noteworthy. In humans, *OTX2* transcripts were largely confined to the retina, optic nerve, sclera, and eyelid, yet protein was detectable at low levels in nearly all ocular tissues. A similar pattern was seen in rats, with transcripts restricted to the retina and low levels in a few additional structures, while protein again showed broader distribution. Such divergence has been reported for other developmental transcription factors and likely reflects mechanisms that buffer fluctuations in transcript availability. In the case of *OTX2*, this buffering may help safeguard critical developmental thresholds, consistent with the incomplete penetrance and variable expressivity of *OTX2* mutations in humans [14].

Our comparative data also revealed quantitative differences: human embryos displayed stronger retinal expression at both transcript and protein levels. These differences may reflect species-specific developmental demands, where *OTX2* activity is required to meet the increased demands of retinal complexity. The stronger expression in humans, even if not statistically significant, supports the notion that *OTX2* function is dosage-sensitive. Clinically, this provides a developmental correlate to the phenotypic variability of human *OTX2* mutations, which range from bilateral anophthalmia to retinal dystrophies [5].

The correlations observed among Otx2 protein levels across multiple tissues further support a model of systemic regulation. Such systemic control may reflect shared upstream regulators, including cross-talk with *PAX6* and *SOX2*, which Hever et al. have implicated in eye malformations [1]. Indeed, *OTX2* and *PAX6* are closely interconnected: loss of *OTX2* leads to upregulation of *PAX6*, particularly in amacrine-like cells that normally express high levels of *PAX6* [7].

Importantly, our study addresses a temporal gap in previous work. Earlier studies have focused largely on the postnatal retina [7], missing the embryonic time points at which gene regulatory networks are first established. By analyzing human and rat embryos, we provide evidence that *OTX2* activity is already compartmentalized during early morphogenesis, with the retina as its primary locus and the eyelid as a secondary site of robust expression.

Importantly, *OTX2* does not act in isolation: Hever and colleagues’ concept of a shared regulatory network is supported by recent findings showing that *OTX2* and *SOX2* directly bind upstream regulatory sequences of Rax, indicating cross-talk among core eye-field transcription factors [32]. This reinforces the notion of systemic coordination among *OTX2*, *SOX2*, and *PAX6* in early ocular morphogenesis.

Moreover, interactions between *PAX6* and *SOX2* in optic cup progenitor regulation highlight that dosage balance and combinatorial expression are critical: perturbation of *PAX6* or *SOX2* levels leads to progenitor fate shifts or abnormal differentiation [33]. In this context, our observed quantitative (though nonsignificant) elevation of *OTX2* in human embryos may reflect an evolutionary “tuning” of dosage thresholds within this intertwined regulatory network, providing a mechanism by which species differences in retinal complexity or developmental constraints are accommodated.

In human embryonic eyes, *PAX6* expression was most pronounced in the retina, whereas other ocular tissues exhibited only low or undetectable transcript levels. Similarly, in rat embryos, *PAX6* transcripts were predominantly localized to the retina, with weak expression detected in the optic nerve, sclera, and eyelid. In contrast, immunohistochemistry revealed a widespread distribution of Pax6 protein in both species. These findings indicate that *PAX6* transcription is spatially restricted, but the protein persists or diffuses more broadly, ensuring functional availability across ocular tissues.

Although humans exhibited stronger retinal *PAX6* expression and broader protein distribution than rats, statistical analysis showed no significant interspecies differences. This suggests that, despite minor variation, Pax6 function in ocular morphogenesis is evolutionarily conserved, consistent with its established role as a dosage-sensitive master regulator of eye development [16,21].

The divergence between *PAX6* gene and protein localization implies that Pax6 protein is stabilized or redistributed into tissues lacking active transcription, coordinating morphogenetic processes across ocular compartments. This supports the view that *PAX6* regulation involves post-transcriptional and post-translational control [16,22,34,35].

Correlation analysis revealed strong interrelationships in Pax6 protein expression across ocular tissues. Perfect correlations were found between the lens, optic nerve, ciliary body, and iris, as well as between the retina and eyelid. Strong associations were also seen between the cornea and extraocular muscles. Such patterns suggest coordinated Pax6 activity among tissues, reflecting shared developmental pathways and its role as an integrator of ocular morphogenesis [21,27].

From a pathological perspective, our data highlight why disruptions in *PAX6* dosage or activity predominantly manifest as ocular abnormalities in humans. Haploinsufficiency leads to classic aniridia, characterized by iris hypoplasia, foveal hypoplasia, and severe visual impairment [5,21]. Structural retinal changes, such as thinning of the macular layers and reduced neuronal density, have also been reported in affected individuals [19]. The broad protein distribution we observed across ocular tissues suggests that Pax6 insufficiency could impair multiple compartments simultaneously, explaining the wide spectrum of ocular phenotypes ranging from aniridia to microcornea, keratitis, and optic nerve anomalies [20]. Our findings of strong inter-tissue correlations in Pax6 protein expression support the hypothesis that dosage effects may propagate across multiple ocular compartments.

Cooperative interactions with transcription factors such as Sox2 further extend Pax6′s developmental role [22,23]. The strong inter-tissue correlations observed may reflect such cooperative networks, ensuring coordinated morphogenesis. Notably, Pax6 continues to function postnatally in the cornea, where it regulates epithelial homeostasis and neural crest migration [18]. The moderate to high embryonic Pax6 protein levels we detected in the cornea and sclera may therefore establish the foundation for later homeostatic functions.

In human embryos, *SOX2* expression was highest in the retina, with weaker signals detected in the optic nerve, sclera, and eyelid. Rat embryos exhibited a similar distribution pattern, although overall transcript levels were lower. By contrast, immunohistochemistry revealed broad protein distribution in both species. In humans, Sox2 protein was most abundant in the retina and eyelid, with moderate to numerous signals also in the cornea and eye muscles, and moderate expression in other ocular compartments. In rat embryos, Sox2 protein was more uniformly expressed across tissues, though at moderate levels. Thus, while both species exhibited restricted *SOX2* transcriptional activity, the protein was widely available, consistent with its role in coordinating morphogenetic processes across ocular tissues.

Although humans displayed higher Sox2 protein abundance in the retina compared with rats, statistical analysis revealed no significant interspecies differences, suggesting that the fundamental role of Sox2 in ocular morphogenesis is evolutionarily conserved. This finding is consistent with extensive evidence that *SOX2* is a dosage-sensitive and conserved transcription factor essential for retinal and ocular development [25,26,28].

Our results align with previous reports that *SOX2* is broadly expressed in the neuroblastic layer of the embryonic retina and later becomes restricted to the inner nuclear layer in the mature eye [27]. In multipotent retinal progenitor cells (RPCs), *SOX2* is indispensable for maintaining proliferative capacity and preventing premature differentiation [25,29,36]. The strong retinal expression we observed is consistent with this role in sustaining RPCs during early ocular development. The widespread protein distribution across other ocular structures may further reflect the requirement for Sox2 in coordinating lens specification, corneal epithelium formation, and optic cup patterning [28,29].

From a pathological perspective, the broad distribution of Sox2 protein provides a mechanistic explanation for the diverse ocular phenotypes associated with *SOX2* mutations in humans. These include anophthalmia, microphthalmia, and structural retinal defects [26,28]. Because Sox2 protein is expressed across nearly all ocular tissues, haploinsufficiency or loss-of-function mutations are likely to impair multiple compartments simultaneously. The strong inter-tissue correlations we observed support the idea that dosage effects propagate across ocular structures, producing complex phenotypes rather than isolated defects.

Our findings also support the hypothesis that Sox2 operates within cooperative transcriptional networks. Previous studies have shown that Sox2 partners with *OTX2* to activate Rax during neural retinal development, while later antagonizing *OTX2* and *PAX6* to refine retinal domain boundaries [28]. Forced *SOX2* expression promotes amacrine cell differentiation while reducing RPC proliferation, in part through Pax6 induction [27]. The correlations observed among ocular tissues in our study may therefore reflect both cooperative and antagonistic interactions with other transcription factors, reinforcing Sox2′s role as a versatile regulator of ocular development.

Importantly, Sox2 remains functionally relevant beyond embryogenesis. In the postnatal and adult retina, Sox2 is maintained in Müller glia, amacrine, and ganglion cells, where it regulates homeostasis and injury responses [25]. With aging, *SOX2*-positive cells decline, correlating with reduced visual capacity and impaired glial function. These findings highlight that Sox2 should not be regarded solely as a progenitor marker but also as a factor required for maintaining retinal integrity throughout life [25,37]. The strong protein expression we observed in the cornea and sclera during embryogenesis may therefore establish the foundation for long-term homeostatic roles [18].

Taken together, our comparative analysis of *OTX2*, *PAX6*, and *SOX2* expression in human and rat embryonic eyes highlights both the conserved nature and the nuanced differences in the regulation of these master transcription factors. In all three cases, transcripts were largely confined to the retina and select posterior structures, whereas the corresponding proteins exhibited broader distribution, highlighting the importance of post-transcriptional and post-translational mechanisms in stabilizing these transcription factors across ocular tissues. The extensive correlations observed among ocular compartments suggest that *OTX2*, *PAX6*, and *SOX2* do not act in isolation but rather as components of an integrated transcriptional network that synchronizes eye development. This coordinated activity helps explain why haploinsufficiency or mutation of any of these genes produces complex and overlapping ocular phenotypes in humans. By providing embryonic-level data across two species, our study not only reinforces the evolutionary conservation of these factors but also emphasizes their dosage-sensitive and cooperative functions in establishing and maintaining ocular architecture. Future studies dissecting their interactions at the molecular level, including with non-coding RNAs and signaling pathways, will be essential for understanding congenital eye malformations and may inform therapeutic approaches aimed at preserving or restoring vision.

However, the results of the present study should be interpreted in light of certain limitations. First, the relatively small sample size may limit the statistical generalizability of the findings. Nevertheless, the material analyzed—human and rat embryonic eye tissues collected at defined stages of early development—is rare and of high scientific value, providing unique insight into species-specific patterns of gene and protein expression during ocular morphogenesis. Second, the analysis was restricted to selected embryonic stages. Because *OTX2*, *PAX6*, and *SOX2* expression is highly dynamic, our data may not capture transient or later developmental changes, particularly those occurring in the postnatal or adult eye. Third, potential technical limitations, including fixation artifacts and possible effects of archival tissue preservation, may have influenced the detection intensity or localization of transcripts and proteins. Finally, although discrepancies between transcript localization and protein distribution were observed, the underlying mechanisms—such as protein stability, translational regulation, or intercellular trafficking—were not directly investigated. Despite these limitations, the present findings provide a valuable baseline for future studies aimed at elucidating the molecular and phenotypic relationships among key transcriptional regulators of ocular development.

The present findings provide a valuable baseline for future studies aimed at elucidating the molecular and phenotypic relationships among key transcriptional regulators of ocular development. Future studies should focus on quantitative and transcriptomic validation of these findings to strengthen spatial and molecular correlations. Approaches such as quantitative co-localization analysis, RNAscope-based high-resolution in situ hybridization, or qPCR confirmation could provide a more detailed assessment of transcript–protein relationships and dynamic expression changes during eye development.

## 4. Materials and Methods

The study was conducted with the approval of the Ethical Committee of Riga Stradiņš University (approval No. 2-PEK-4/441/2025; 28 February 2025). All procedures were carried out at the Institute of Anatomy and Anthropology, Latvia. A total of six human embryonic eye specimens and three rat embryonic eye specimens were obtained from the institute’s historical paraffin blocks archival collections.

Human specimens represented gestational ages between six and nine weeks, comprising three samples at 6–7 weeks, two samples at 7–8 weeks, and one sample at 8–9 weeks of gestation. These correspond approximately to Carnegie stages (CS) 18–23, covering late embryonic development through the transition into the early fetal period. During this window, optic cup morphogenesis, lens differentiation, and early retinal layering are actively progressing, providing a key developmental context for the present analysis. Rat specimens represented developmental stages between two and three weeks of gestation. All samples were processed and systematically analyzed for gene and protein expression patterns.

### 4.1. Chromogenic In-Situ Hybridization (CISH)

To enable the visualization of putative messenger RNA (mRNA) transcripts of the candidate genes, chromogenic in situ hybridization (CISH) was employed [38]. For this purpose, *OTX2*, *PAX6*, and *SOX2* probe sets (Empire Genomics, New York, NY, USA) were used, consisting of digoxigenin-labeled DNA sequences specifically designed to hybridize with the corresponding mRNA transcripts within tissue sections.

Pretreatment of tissue sections was performed in accordance with established laboratory protocols. For probe application, 10 μL of the digoxigenin-labeled probe was carefully dispensed onto each pretreated specimen using a calibrated micropipette. The sections were subsequently overlaid with a 22 × 22 mm coverslip and placed on a hot plate at 79 °C for 5 min to achieve denaturation. Hybridization was then carried out by transferring the slides to a controlled-humidity chamber, where they were incubated overnight at 37 °C under conditions preventing desiccation. On the following day, coverslips were removed by sequential immersion of the slides in SSC and TBS wash buffers. Thereafter, the specimens were subjected to the remaining steps of the CISH protocol in accordance with the manufacturer’s instructions. The slides were then washed in cold running water for 2 min, dehydrated in 96% ethanol, and cleared in xylene. Finally, coverslips were mounted, and probe hybridization signals were evaluated by brightfield microscopy [39,40].

Specimens were evaluated using a semi-quantitative scoring approach [41]. Hybridization signals, visualized as turquoise-colored dots, were assessed at 400× magnification under a light microscope (Leica DC 300F, Leica Biosystems, Richmond, VA, USA). All tissue samples were independently examined by two experienced morphologists. For image processing, visual illustration, and analysis Image-Pro Plus software, version 7.0, Media Cybernetics, Rockville, MD, USA) was used.

### 4.2. Biotin–Streptavidin Immunohistochemistry (IHC)

The biotin–streptavidin biochemical method was used for the detection of Otx2, Pax6, and Sox2 (Table 1).

Prepared tissue sections (deparaffinized, rehydrated, and cleared) were rinsed in TRIS wash buffer (Diapath, Martinengo, Italy) for 10 min, followed by antigen retrieval through boiling in EDTA buffer (AppliChem, Darmstadt, Germany) for 10 min. Sections were subsequently cooled to 65 °C and again rinsed in TRIS wash buffer. Endogenous peroxidase activity was blocked with 3% hydrogen peroxidase solution (Dako, Glostrup, Denmark). Primary antibodies were diluted in Antibody Diluent (Cell Marque™, Rocklin, CA, USA) and applied to the sections, which were incubated for 2 h in accordance with the manufacturer’s instructions. After washing in TRIS buffer, signal detection was performed using the HiDef Detection™ HRP polymer system (Cell Marque™, Rocklin, CA, USA) for rabbit antibodies, as recommended by the supplier. Sections were then incubated sequentially with a biotinylated secondary antibody for 30 min and a tertiary antibody, each followed by a 10-min rinse in TRIS buffer. Immunoreactivity was visualized using DAB+ chromogen (DAB Substrate Kit, Cell Marque™, Rocklin, CA, USA), with incubation at room temperature for 10 min producing a brown precipitate in positive structures. Slides were washed in distilled water, counterstained with hematoxylin for 2 min, dehydrated through graded ethanol, and cleared in carboxylol and xylene. Appropriate positive and negative immunohistochemical controls were included for each sample. Negative controls included omission of primary antibody, isotype-matched rabbit IgG at equal concentration, peptide pre-absorption (where available), biotin/streptavidin system controls with avidin–biotin blocking and reagent-only runs, non-expressing control tissues, and internal anatomical negatives; none showed specific DAB reactivity.

Immunoreactive structures were evaluated at 200× magnification under a light microscope (Leica DC 300F, Leica Biosystems, Richmond, VA, USA). The evaluation was completed by two researchers to avoid subjectivity. For image processing, visual illustration, and analysis Image-Pro Plus software (Media Cybernetics, Rockville, MD, USA) was used.

### 4.3. Semi-Quantitative Scoring Method

A semi-quantitative scoring system was applied to assess the quantity and distribution of gene- and protein-positive structures. For CISH, turquoise-colored hybridization signals were evaluated, whereas for IHC the number of positively stained cells was recorded within light microscopic vision fields. The scoring scale was defined as follows: 0—no positive structures; 0/+—occasional positive structures; +—few positive structures; +/++—few to moderate positive structures; ++—moderate positive structures; ++/+++—moderate to numerous positive structures; +++—numerous positive structures; +++/++++—numerous to abundant positive structures; ++++—abundant positive structures (Table 2) [41].

### 4.4. Statistical Analysis

Statistical analyses were performed using the Statistical Package for the Social Sciences (SPSS), version 27.0 (IBM Corp., Armonk, NY, USA). The results of the semi-quantitative scoring system were converted into numerical values for statistical processing as follows: no positive structures = 0; occasional positive structures = 0.5; few positive structures = 1.0; few to moderate positive structures = 1.5; moderate positive structures = 2.0; moderate to numerous positive structures = 2.5; numerous positive structures = 3.0; numerous to abundant positive structures = 3.5; abundant positive structures = 4.0.

Nonparametric statistical methods were applied for data analysis. Differences between human and rat eye samples were assessed using the Mann–Whitney U test [42], while paired comparisons within the same specimens (e.g., between expression levels of different markers) were evaluated using the Wilcoxon signed-rank test [43]. Correlations between variables were evaluated using Spearman’s rank correlation coefficient (r_s_) [44]. The strength of correlation was interpreted as follows: r_s_ = 0.80–1.00, very strong; r_s_ = 0.60–0.79, strong; r_s_ = 0.40–0.59, moderate; r_s_ = 0.20–0.39, weak; and r_s_ < 0.20, very weak. A *p*-value ≤ 0.05 was considered indicative of statistical significance for all tests.

## 5. Conclusions

1. *OTX2* transcript restriction to the retina underpins the structural differentiation of photoreceptors and bipolar cells, while its broader protein distribution suggests a stabilizing role in preserving retinal architecture and adjacent ocular tissues.

2. Although *PAX6* transcription is largely retinal, its widespread protein distribution across ocular tissues highlights its function as a central coordinator of eye morphogenesis.

3. Strong retinal *SOX2* transcription supports the maintenance of multipotent retinal progenitors, whereas its broad protein expression across the cornea, sclera, and optic cup indicates a structural role in shaping diverse ocular compartments.

Together, these expression patterns provide a molecular framework that may inform the optimization of stem cell–derived ocular organoid models and guide strategies for retinal tissue engineering.

## Figures and Tables

**Table 1:** Description and characteristics of the antibodies.

Antibody	Characteristics	Dilution	Catalogue No.	Manufacturer
Otx2	Polyclonal rabbit	1:50	orb306089	Biorbyt (Durham, NC, USA)
Pax6	Polyclonal rabbit	1:200	orb612166	Biorbyt (Durham, NC, USA)
Sox2	Polyclonal rabbit	1:200	orb11398	Biorbyt (Durham, NC, USA)

Abbreviations: Otx2—Orthodenticle Homeobox 2, Pax6—Paired Box 6, Sox2—SRY-box Transcription Factor 2.

**Table 2:** Semi-quantitative scoring system for CISH and IHC evaluation.

Score	Description of Positive Structures
0	No positive structures
0/+	Occasional positive structures
+	Few positive structures
+/++	Few to moderate positive structures
++	Moderate positive structures
++/+++	Moderate to numerous positive structures
+++	Numerous positive structures
+++/++++	Numerous to abundant positive structures
++++	Abundant positive structures

## Data Availability

The original contributions presented in this study are included in the article and Supplementary Materials. Further inquiries can be directed to the corresponding author.

## Supplementary Materials

The following supporting information can be downloaded at: https://www.mdpi.com/article/10.3390/ijms262210845/s1.

## Abbreviations

The following abbreviations are used in this manuscript:

CISH	Chromogenic in situ hybridization
IHC	Immunohistochemistry
*OTX2*	Orthodenticle homeobox 2 gene
*PAX6*	Paired box 6 gene
*SOX2*	SRY-box transcription factor 2 gene
Otx2	Orthodenticle homeobox 2 protein
Pax6	Paired box 6 protein
Sox2	SRY-box transcription factor 2 protein
