# Supplementary material for: Analysis of OTX2, PAX6, and SOX2 Gene and Protein Expression Patterns in Ocular Development of Human and Rat Embryos
Source: Int J Mol Sci. 2025 Nov 8;26(22):10845. doi: 10.3390/ijms262210845 (PMC12652490; doi:10.3390/ijms262210845)
Supplement: Supplementary file 1 [file ijms-26-10845-s001.zip › ijms-3946087-supplementary.pdf]

Supplementary Table S1. Median semi-quantitative score of the results obtained from CISH and IHC.

Localization	Chromogenic In-Situ Hybridization (CISH)			Immunohistochemistry (IHC)		
	**OTX2**	**PAX6**	**SOX2**	**Otx2**	**Pax6**	**Sox2**
			Human Embryo			
Lens	0	0	0	1+	3+	2+
Optic Nerve	1.75+	1.25+	1.5+	1+	3+	2+
Ciliary Body	0	0	0	1+	3+	2+
Iris	0	0	0	1+	3+	2+
Cornea	0	0	0	1+	2.75+	2.75+
Sclera	1.75+	1.25+	1.5+	1+	2+	2+
Choroid	0	0	0	1+	2+	2+
Retina	2.75+	2.25+	2.5+	4+	3+	3+
Eye muscles	0	0	0	1+	3+	2.5+
Eyelid	1.75+	1.25+	1.5+	4+	3+	3+
			Rat Embryo			
Lens	0	0	0	1+	2+	2+
Optic Nerve	1+	1+	1+	1+	2+	2+
Ciliary Body	0	0	0	1+	2+	2+
Iris	0	0	0	1+	2+	2+
Cornea	0	0	0	1+	2+	2+
Sclera	1+	1+	1+	1+	2+	2+
Choroid	0	0	0	1+	2+	2+
Retina	2+	2+	2+	3+	3+	2+
Eye muscles	0	0	0	0	3+	2+
Eyelid	1+	1+	1+	3+	3+	2+

Abbreviations: OTX2 – Orthodenticle Homeobox 2 gene, PAX6 – Paired Box 6 gene, SOX2 – SRY-box Transcription Factor 2 gene, Otx2 – Orthodenticle Homeobox 2 protein, Pax6 – Paired Box 6 protein, Sox2 – SRY-box Transcription Factor 2 protein.
